# On the Chemical and Biological Characteristics of Multifunctional Compounds for the Treatment of Parkinson’s Disease

**DOI:** 10.3390/antiox12020214

**Published:** 2023-01-17

**Authors:** Olimpo García-Beltrán, Pamela J. Urrutia, Marco T. Núñez

**Affiliations:** 1 Facultad de Ciencias Naturales y Matemáticas, Universidad de Ibagué, Carrera 22 Calle 67, Ibagué 730002, Colombia; 2 Centro Integrativo de Biología y Química Aplicada (CIBQA), Universidad Bernardo O’Higgins, General Gana 1702, Santiago 8370854, Chile; 3 Faculty of Medicine and Science, Universidad San Sebastián, Lota 2465, Santiago 7510157, Chile; 4 Faculty of Sciences, Universidad de Chile, Las Palmeras 3425, Santiago 7800024, Chile

**Keywords:** Parkinson’s disease, iron dyshomeostasis, clinical trials, multifunctional drugs

## Abstract

Protein aggregation, mitochondrial dysfunction, iron dyshomeostasis, increased oxidative damage and inflammation are pathognomonic features of Parkinson’s disease (PD) and other neurodegenerative disorders characterized by abnormal iron accumulation. Moreover, the existence of positive feed-back loops between these pathological components, which accelerate, and sometimes make irreversible, the neurodegenerative process, is apparent. At present, the available treatments for PD aim to relieve the symptoms, thus improving quality of life, but no treatments to stop the progression of the disease are available. Recently, the use of multifunctional compounds with the capacity to attack several of the key components of neurodegenerative processes has been proposed as a strategy to slow down the progression of neurodegenerative processes. For the treatment of PD specifically, the necessary properties of new-generation drugs should include mitochondrial destination, the center of iron-reactive oxygen species interaction, iron chelation capacity to decrease iron-mediated oxidative damage, the capacity to quench free radicals to decrease the risk of ferroptotic neuronal death, the capacity to disrupt α-synuclein aggregates and the capacity to decrease inflammatory conditions. Desirable additional characteristics are dopaminergic neurons to lessen unwanted secondary effects during long-term treatment, and the inhibition of the MAO-B and COMPT activities to increase intraneuronal dopamine content. On the basis of the published evidence, in this work, we review the molecular basis underlying the pathological events associated with PD and the clinical trials that have used single-target drugs to stop the progress of the disease. We also review the current information on multifunctional compounds that may be used for the treatment of PD and discuss the chemical characteristics that underlie their functionality. As a projection, some of these compounds or modifications could be used to treat diseases that share common pathology features with PD, such as Friedreich’s ataxia, Multiple sclerosis, Huntington disease and Alzheimer’s disease.

## 1. Introduction

PD is a slowly progressive neurodegenerative disorder that affects 2–3% of the population over 65 years of age. The immediate cause of PD is the loss of dopaminergic neurons in the substantia nigra pars compacta (SNpc). These neurons project into the striatum, where they interact with two populations of the spiny projection neurons that form the direct and indirect pathway of the basal ganglia, whose coordinated activity is essential for movement control [1]. In PD patients, the decrease in dopamine signaling to the striatum causes the hypo- and brady-kinetic symptoms of the disease [2].

Among neurological disorders, PD is the world’s fastest-growing prevalence, with disability and deaths increasing from 2.5 million people affected globally in 1990 to 6.1 million in 2016 [3]. Driven mostly by aging, this number is projected to exceed 12 million affected persons worldwide by the year 2040 [4]. This pathology also has a high financial impact, reporting a total economic burden of $51.9 billion, annually, in the USA [5].

Traditionally, pharmacological PD treatments aim to compensate for the decreased content of dopamine derived from the death of substantia nigra dopaminergic neurons, either by L-DOPA supplementation, the inhibition of Mono Amino Oxidases (MAO) or by agonists of the dopamine receptors. These treatments improve the quality of life of the patients but do not stop neuronal death and, in the long term, loose effectivity and generate toxicity [6,7,8].

Pathophysiological events associated with the death of SNpc dopaminergic neurons include mitochondrial dysfunction, which results in oxidative stress and decreased ATP production; iron dyshomeostasis and its associated free radical production, which results in lipoperoxidation and ferroptotic cell death; α-synuclein aggregation, which results in impaired proteasomal protein clearance; and mitochondrial dysfunction and inflammatory conditions, which accelerate the neurodegenerative process. In consideration of the multiplicity of factors involved in this neurodegenerative process, therapeutic interventions addressing a single target do not hold much promise of long-term success, as has been experienced by finalized clinical trials. Accordingly, a multi-target approach that deal with several aspects of the neurodegenerative process simultaneously may be a better approach to stop the progress of the disease [9,10,11].

An ideal drug for the treatment of PD should have an effect on the main pathophysiological events found in PD. Arguably, an ideal drug should have: the capacity to quench free radicals; the capacity to disrupt or impede α-synuclein aggregation; iron chelation capacity; the capacity to decrease inflammatory conditions; and have a mitochondrial destination, which is the center of iron-reactive oxygen species interaction. Additionally, an ideal multifunctional drug should have a MAO-B inhibitory capacity to increase the intracellular dopamine content and be selective for dopaminergic neurons to impede unwanted effects in other cell types. To facilitate treatment and increase compliance, the drug should be orally effective, which comprises both good intestinal absorption and blood-brain barrier permeability.

Here, we review the processes that underlie neuronal death in PD and the surge of multifunctional compounds that are effective in the neutralization of two or more aspects of the pathological process.

## 2. Mitochondria-Associated Events in PD Neurodegeneration

The mitochondrion is the center of the oxidative metabolism and the main site of continuous reactive oxygen species (ROS) production. Similarly, it is crucial in health and its dysfunction contributes to the pathogenesis of several diseases, including PD (reviewed in [12,13,14]).

Mitochondrial dysfunction is a key factor in SNpc neuron death. Indeed, diminished mitochondrial complex I activity has been widely observed in postmortem tissue from PD patients. In addition, several familial PD-associated genes encode the proteins involved in the maintenance of mitochondrial function and the degradation of damaged mitochondria [15]. Moreover, toxins that inhibit mitochondrial complex I are widely used to generate experimental models of PD.

**Figure 1:**
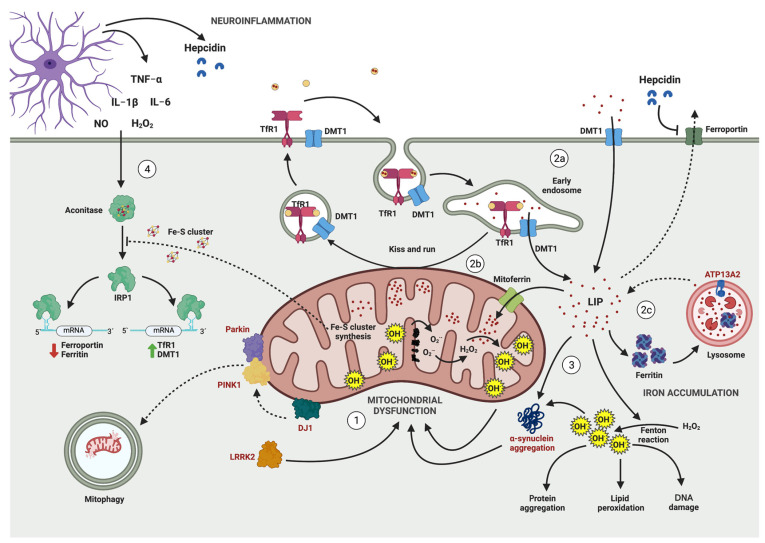
**Interplay between neuroinflammation, mitochondrial dysfunction, iron accumulation, and α-synuclein aggregation in PD.** Mitochondrial dysfunction takes place downstream of PD-associated genes mutations (encoded proteins in red), which induce mitochondrial ROS (mtROS) generation and oxidative damage (**1**). Mitochondrial dysfunction impairs Fe-S cluster synthesis prompting iron accumulation through IRE/IRP dysregulation. Iron uptake is mediated by TfR1 and DMT1, which are increased in PD, while ferroportin, which mediates iron export, is decreased, causing iron accumulation and an increased iron labile pool (**2a**). Iron is transported to mitochondria by mitoferrin or by an endocytic mechanism named “kiss and run”. Iron inside of mitochondria is used for Fe-S cluster synthesis. However, mitochondrial iron accumulation catalyzes the Fenton reaction, triggering mtROS production (**2b**). Iron excess is stored in ferritin, which is released by lysosome-mediated ferritin degradation, which is also altered in PD (2c). Cytoplasmic iron accumulation triggers hydroxyl radical production, which promotes lipid peroxidation and DNA damage and α-synuclein aggregation, which further enhances mitochondrial dysfunction (**3**). Neuroinflammation also alters iron homeostasis (**4**). Pro-inflammatory cytokines, NO and H_2_O_2_ increases DMT1 and TfR1 protein expression and decreases ferroportin and ferritin levels through IRP1-dependent and independent mechanisms.

Downstream, mitochondrial dysfunction is interlinked with other pathophysiological events associated to PD neurodegeneration, including iron accumulation, oxidative stress, α-synuclein aggregation and neuroinflammation (Figure 1). It follows that the multiplicity of factors comprising PD neuronal death should be considered in the planning of effective treatments for the disease.

### 2.1. Mitochondrial Dysfunction and ROS in PD

The main functions of mitochondria are the production of ATP, the regulation of Ca^2+^ homeostasis, heme biosynthesis, the formation and export of iron-sulfur (Fe-S) clusters, cell division control and the control of cell death via apoptosis [16]. In addition, mitochondria are the major intracellular source of ROS and are the site of the Krebs cycle that translate the products of the catabolism of carbohydrates, lipids, and proteins into reduced nicotinamide adenine dinucleotide and flavin adenine dinucleotide, which are substrates of the electron transport chain [17].

The parkinsonism triggered by the accidental injection of the selective inhibitor of mitochondrial complex I, 1-methyl-4-phenyl-1, 2, 3, 6- tetrahydrodropyridine (MPTP), provides the first evidence that mitochondria have a main role in PD pathogenesis. [18,19]. Subsequently, several studies have revealed that other complex I inhibitors, such as Rotenone, pyridaben, trichloroethylene and fenpyroximate, also induce dopaminergic neurodegeneration in flies, humans and rodents, reinforcing the idea that mitochondrial dysfunction is an essential characteristic in idiopathic PD pathogenesis. These toxins cause alterations in the mitochondrial electron activity, triggering chronic ROS production, increasing the activity of mitochondrial nitric oxide synthase and reducing ATP synthesis. These events are observed in the SNpc neurons, skeletal muscle and platelets of PD patients, where the activity of complex I is impaired and several subunits of complex I are oxidatively damaged [20].

In addition, structural changes in complex I triggered by a deficiency of apoptosis-inducing factors make the dopaminergic neurons more vulnerable to neurotoxins [21]. Moreover, the deletion of NADH:ubiquinone oxidoreductase subunit S4 (NDUFS4), an accessory subunit involved in complex I assembly and stability, causes reactive gliosis in the brainstem and produces motor symptoms [21]. The conditional deletion of NDUFS4 in mice dopaminergic neurons causes a decline in dopamine levels in the striatum and amygdala and non-motor manifestations, such as anxiety and cognitive defects [22], conditions resembling a pre-symptomatic stage of PD. Nonetheless, the importance of complex I inhibition in the death of dopaminergic neurons was recently questioned due to the conflicting results obtained in the Ndfus4 null mice. Studies show that Ndufs4-deficient dopaminergic neurons are equally as sensitive as wild-type neurons to neurotoxins such as rotenone, paraquat, MPP^+^ [23] or Maneb [24], suggesting that mitochondrial complex I inhibition is not required for the dopaminergic neuron death induced by these toxins. Additionally, mice with conditional Ndufs4 KO, specifically in in dopaminergic neurons, did not show enhanced neurodegeneration or sensibility to MPTP [25]. The absence of spontaneous neurodegeneration in the Ndufs4 knockout mouse has been confirmed by another independent group, although this mouse is more vulnerable to MPTP treatment [26]. These divergent observations could be explained by differences in penetrance caused by a remnant activity of complex I in the Nudfs4 KO mouse [27] or by unknown compensatory mechanisms to Ndufs4 deficiency.

Notwithstanding, it is safe to establish that mitochondrial dysfunction in SNpc neurons is intimately linked to many of the pathognomonic signs of idiopathic PD. This concept is reinforced by the genetic mutations that result in familial forms of PD.

The loss-of-function mutations of PTEN-induced kinase 1 (*PINK1*) or *Parkin* are responsible for autosomal recessive early-onset PD. PINK1 and Parkin proteins participate in mitochondrial quality control by regulating mitophagy [28]. *PINK1* encodes a serine/threonine-protein kinase that is localized in the outer mitochondrial membrane (OMM) or mitochondrial intramembrane space (IMS), depending on the membrane potential. In polarized mitochondria, PINK1 is imported into the IMS, where it is degraded by proteases [29]. In contrast, mitochondrial depolarization deactivates PINK degradation, leading to its accumulation in the OMM. PINK1 recruits Parkin, an E3 ubiquitin ligase, which ubiquitinates selected proteins on the mitochondrial surface, generating a signal for mitochondrial degradation [30,31]. Therefore, normal low levels of PINK1 prevent mitophagy in healthy mitochondria, while the activation of the PINK1/Parkin pathway by membrane depolarization allows for the removal of damaged mitochondria. Accordingly, several studies show that rodents and cells lacking Parkin or PINK1 have reduced complex I or complex IV activity, decreased mitochondrial integrity, lower respiratory capacity and synthesis of ATP and higher ROS production [32,33,34,35,36,37].

Mutations in the gene encoding the DJ-1 protein are associated with autosomal recessive early-onset parkinsonism [38], which is found in approximately 1% of PD familial cases. DJ-1 is a peptidase of the C56 family, whose functions are not completely understood. DJ-1 is required for a normal life span, motor function and neuronal resistance to oxidative damage [39,40,41,42], and it is implicated in mitochondrial biology. Defects in DJ-1 expression alter mitochondrial morphology and function [43], increases ATP production and ROS production, and opens the mitochondrial permeability transition pore [41,44]. Interestingly, in response to mitochondrial stress and oxidative damage, DJ-1 translocates from the cytosol to the mitochondria [45,46], where it can rescue the PINK loss-of-function phenotype [41], suggesting that it also participates in the elimination of damaged mitochondria. Recently, it was reported that DJ-1 maintains dopaminergic cell metabolism and neuronal growth by regulating the ATP synthase protein components [47].

Mutations in the *SNCA* gene are responsible for autosomal dominant forms of PD. The *SNCA* gene encodes the α-synuclein, a protein predominantly located in the synaptic terminals, where it is thought to play a role in vesicular packaging and trafficking [48,49]. As it has small amounts of a secondary structure, α-synuclein has a high propensity to form aggregates [50]. Aggregated α-synuclein is the main structural building block for the formation of Lewy bodies, a pathological hallmark of PD [51]. Lewy bodies are found in the remaining DA neurons of the SNpc, as intraneuronal eosinophilic inclusions are positive for α-synuclein and ubiquitin. Additionally, in most PD cases, Lewy neurites are found in the amygdala and striatum, which are neurites containing granular material and α-synuclein filaments (reviewed in [52])

The propensity of α-synuclein to form aggregates can be influenced by several factors, including missense mutations (best known: A53T, E46K, A30P) [53,54], environmental toxins [54,55] and high metal concentration, including aluminum, copper(II), iron(III), cobalt(III), and manganese(II) [56].

The α-synuclein aggregates impact mitochondrial function. α-synuclein binds to the OMM, inhibiting mitochondrial protein import, triggering mitochondrial senescence and increasing mitochondrial ROS production [57]. Additionally, α-synuclein aggregates reduce the mitochondrial membrane potential, inhibit complex I activity [58] and induce mitochondrial fragmentation, which can be rescued by PINK1, parkin or DJ-1 overexpression [59,60,61].

Leucine rich repeat kinase 2 (LRRK2) is a large multidomain protein that has a central catalytic tri-domain with GTPase and kinase activities surrounded by a series of protein–protein interaction domains [62]. Mutations in the *LRRK2* gene are responsible for autosomal dominant forms of familial PD [63]. Several studies show that LRRK2 regulates several cellular processes, including endo-lysosomal vesicle trafficking, mitochondrial homeostasis, autophagy, neurite outgrowth, cytoskeletal maintenance and immune system function (reviewed in [64]).

LRRK2 is localized in several subcellular compartments, including the mitochondrion [65]. Its overexpression generates mitochondrial fragmentation, along with the increased expression of the mitochondrial dynamin-like protein (DLP) and ROS production [66]. Moreover, human fibroblasts or induced pluripotent stem cells (iPSCs)-derived neurons from PD patients carrying the *LRRK2* G2019S mutant (the most frequently occurring mutation) show impaired mitochondria function [67,68], increased ROS production [67], increased rotenone sensibility [69] mitochondrial DNA damage [70], increased mitochondrial fragmentation [71,72] and excessive mitophagy [73,74].

Mutations in the *ATP13A2* gene, which encodes for a lysosomal P-type ATPase, cause a rare autosomal recessive parkinsonism known as Kufor Rakeb Syndrome (KRS) [75]. Patients with KRS show an increased iron deposition in the basal ganglia [76]. ATP13A2 plays an important role in the maintenance of iron homeostasis as lysosomes can act as reservoirs of redox-reactive ferrous iron [77]. The loss of ATP13A2 activity disrupts the lysosome’s ability to store iron excess, reducing dopaminergic neuronal cell survival [78]. In addition, ATP13A2 overexpression reduces iron-induced lysosomal membrane permeabilization and cytotoxicity, triggering an enlargement of the lysosomes and late endosomes [79]. Lysosomes are essential for autophagy, where the autophagic clearance of dysfunctional mitochondria represents a crucial element of mitochondrial quality control. Accordingly, ATP13A2-deficiency induces mitochondrial dysfunction [80,81,82] and increases mitochondrial ROS production [82].

Thus, it is safe to conclude that mitochondrial dysfunction is a central feature to both idiopathic and most familial cases of PD.

### 2.2. Mitochondrial Dysfunction and Iron Homeostasis in PD

As mentioned previously, the mitochondrion plays a central role in the biology of Fe-S clusters as it holds the assembly machinery responsible for their synthesis (reviewed in [83]). Fe-S cluster synthesis also occurs in the cytoplasm, albeit at a minor scale, and depends on a sulfur-containing factor generated in mitochondria.

Eukaryotic cells have, approximately, between 50 and 70 different Fe-S clusters-containing proteins, which support a wide array of biological processes through redox-dependent and independent mechanisms. Mitochondrial Fe-S proteins take part in the electron transport chain (complexes I-III), lipid β-oxidation, heme biosynthesis and the citric acid cycle (aconitase). On the other hand, cytosolic and nuclear Fe-S proteins include several DNA polymerases, DNA helicases and glycosidases, proteins required for transfer RNAs modification, ribosome biogenesis and function, cellular iron homeostasis maintenance (IRP1), antiviral defense and finally kinesin family member 4A, a molecular motor associated to chromosome segregation during mitosis (reviewed in [84]).

Beyond its essentiality for Fe-S cluster synthesis, iron excess is linked to cellular death, causing protracted cellular oxidative stress through the iron-mediated non-enzymatic catalytic conversion of H_2_O_2_ and O_2_^•−^ into the highly reactive hydroxyl radical as a result of Fenton and Haber-Weiss chemistry [85,86]. Therefore, iron homeostasis must be tightly controlled to satisfy iron requirements while avoiding iron toxicity, particularly in the mitochondrion, as this organelle, with its high content of iron and ROS, allows for a favorable environment for iron-mediated oxidative damage.

The iron transport protein transferrin (Tf), present in serum/cerebrospinal fluid, plays an essential role in cellular iron uptake. Tf possesses two high affinity iron (III)-binding sites. Iron-loaded Tf is endocytosed by binding to transferrin receptor-1 (TfR1). Ferric ions from endocytosed Tf dissociate in the acidic environment of the endosome and are subsequently reduced to ferrous ions and released into the cytosol by divalent metal transporter-1 (DMT1) [87]. This pool of chelatable and redox-active iron is known as the cytosolic labile iron pool (cLIP). Iron in the cLIP is distributed to three destinations: (i) to mitochondria, for the synthesis of Fe-S clusters or heme group; (ii) to ferritin, a cytosolic iron storage protein; or (iii) transported back to the extracellular medium through the iron exporter, Ferroportin 1 (FPN1). The iron stored in ferritin become bioavailable after ferritin lysosomal degradation [88,89,90].

Changes in cell iron status (iron overload or depletion) lead to compensating translational changes of the iron homeostasis-related proteins, mediated by the iron responsive element (IRE)/Iron regulatory protein (IRP) system. Two IRPs isoforms, IRP1 and IRP2, modulate the expression of proteins by binding to conserved stem-loop structures, named IREs, in the mRNA untranslated regions (UTRs). The regulatory outcome depends upon the position and context of the IRE in the mRNA: IRP binding to a 5′ UTR IRE represses translation, whereas IRP interaction to a 3′ UTR IRE can indirectly stimulate translation through the suppression of mRNA degradation. In iron-deficient cells, IRPs selectively bind IRE at 5´ UTR of the mRNA coding for ferritin and FPN1 and to 3`UTR of the mRNA coding for TfR1 and DMT1, promoting iron uptake. When iron is in excess, IRP2 is degraded, and IRP1 apoprotein binds a [4Fe-4S] cluster by a successive transfer of two [2Fe-2S] clusters to become a functioning cytosolic (c)- aconitase, suppressing its RNA-binding activity. Diminished IRPs binding to the IREs promotes ferritin and FPN1 synthesis and the TfR1 and DMT1 mRNAs are degraded by nucleases.

Iron is imported into mitochondria by two mechanisms. The first mechanism is named “endosome kiss and run”, by which iron bypasses cytoplasmic transport, and is instead released into the mitochondria by a transient physical contact between the endosome and outer mitochondrial membrane [91]. Recently, it was reported that DMT1 participates in this mechanism by regulating the interactions between mitochondria and early endosomes [92]. In the second mechanism, a mitochondrial iron importer mitoferrin delivers iron to mitochondria from the cLIP [93,94,95]. This mechanism is also regulated by IRP1, as IRP1 stimulates mitoferrin expression, enhancing the mitochondrial iron import [96].

Several studies show elevated iron content in glial cells and dopaminergic neurons of the SNpc of PD patients [97,98,99,100]. This increased iron is thought to contribute to neuronal death by mediating the production of hydroxyl radicals and by promoting the fibril formation of α-synuclein aggregation and fibrillation [101]. Neuroprotection, achieved by pharmacological or genetic chelation of iron in animal models of PD, supports its role in neuronal degeneration (discussed below).

Interestingly, dysregulation on cellular iron homeostasis is observed in cellular and animal models of PD that are based in mitochondrial complex I inhibition. In MPTP intoxicated animals, elevated DMT1 expression in the ventral mesencephalon takes place with iron accumulation, oxidative stress and dopaminergic neurons loss. In neuroblastoma cell lines, MPP^+^ (the active metabolite of MPTP) treatment also induces the up-regulation of DMT1, generating iron accumulation and ROS [102]. Moreover, treatment with rotenone increases TfR1 and DMT1 levels and decreases FPN1 levels, together with an increment in iron uptake and increase in the cLIP [103]. Complex I inhibition also increases cysteine oxidation and lipid peroxidation [103]. These changes were accompanied by an increase in IRP1 activity due to a decrease in Fe-S cluster synthesis [104]. By contrast, IRP1 knockdown induces higher ferritin levels and a lower LIP, increases resistance to cysteine oxidation and decreases lipid peroxidation, protecting it from complex I inhibition-induced cell death [103]. These results support the concept that mitochondrial dysfunction results in IRP1 activation, triggering iron accumulation and cell death.

### 2.3. Mitochondrial Dysfunction and Neuroinflammation in PD

Chronic neuroinflammation is one of the hallmarks of PD pathology. Several studies using postmortem tissue from PD patients show a dramatic proliferation of reactive amoeboid macrophages and microglia in the SNpc. Macrophage proliferation is accompanied by a high expression of pro-inflammatory cytokines, such as tumor necrosis factor-α (TNF-α), interleukin-1β (IL-1β), interferon-gamma (IFN-γ) and interleukin-6 (IL-6), as well as the production of reactive oxygen and nitrogen species by glial cells (reviewed in [105,106]). This increase in pro-inflammatory cytokines is also observed in the cerebrospinal fluid and basal ganglia of patients with PD [107]. These features are reproduced in several animal models of PD, including 6-hydroxydopamine-, MPTP-, rotenone- or LPS-injected rodents (reviewed in [108]).

Recently, it was proposed that ROS derived from dysfunctional mitochondria (mtROS) can activate the NOD-, LRR- and pyrin domain-containing protein 3 (NLRP3) inflammasome and the secretion of pro-inflammatory cytokines in PD [109]. These observations may help us to comprehend the often-observed association between mitochondrial damage and inflammation in neurodegenerative diseases, including PD (reviewed in [110]).

The inflammasome is a cytoplasmic multiprotein complex composed by a sensor protein, named the pattern-recognition receptor (PRR), and an inflammatory caspase. In some inflammasomes, both the PRR and the inflammatory caspase are connected by an adapter protein. Several PRR families have been described, with the best-characterized members being the Toll-like receptor (TLR) family, RIG-I-like receptors (RLRs), nucleotide-binding domain leucine-rich repeat-containing proteins (NLRs), C-type lectin receptors (CLRs) and AIM2-like receptors (ALRs).

Many pathogen-associated molecular patterns (PAMPs) and damage-associated molecular patterns (DAMPs) can activate the inflammasomes by PRR binding, stimulating both the canonical and non-canonical pathway. The canonical pathway depends on caspase 1 activation, whereas the non-canonical pathway depends on caspase 11 (caspase 4 or 5 in humans) or caspase 8 activation. Both converge in the production of IL-1β, IL-18 and gasdermin D, which induces pyroptosis by forming pores in the cell plasma membrane (reviewed in [111]).

The NLRP3 inflammasome primarily sense exogenous signals, but also respond to endogenous signals, including mitochondrial dysfunction and ROS (reviewed in [112]). The first study linking mitochondrial dysfunction with NLRP3 inflammasome activation showed that mtROS, generated downstream of the inhibition of the mitochondrial respiratory chain, can activate the NLRP3 inflammasome canonical pathway [113]. Accordingly, ROS induces thioredoxin-interacting protein binding to NLRP3, which is essential for NLRP3 inflammasome activation [114].

In addition, mtROS can damage the mitochondrial genome and open the mitochondrial permeability transition pore, allowing for the escape of oxidized mitochondrial DNA (mtDNA) to the cytosol, acting as damage-associated molecular patterns and, accordingly, activating TLR9 [115], the NLRP3 inflammasome [116], the AIM2 inflammasome [117] and the cytosolic cyclic GMP-AMP synthase (cGAS)-stimulator of the interferon genes (STING) pathway [118]. The latter activates the NF-κB transcriptional factor, triggering the production of type I IFN and the expression of inflammatory cytokines, such as tumor necrosis factor (TNF), IL-1β and IL-6 [119]. Interestingly, the loss of STING prevented SNc dopaminergic neuron death and motor deficits in a Parkin-null mouse that also expressed a proofreading-defective mtDNA polymerase (accumulating mtDNA mutations with aging), suggesting that inflammation mediates the neurodegeneration in this model [109]. Mitochondrial transcription factor A contributes to eliminating damaged mtDNA molecules bearing apurinic/apyrimidinic (abasic) sites [120]. Moreover, TFAM KO mice develop a PD-like neurodegeneration [121], and Idiopathic PD patients displays increased levels of abasic sites together with a reduced expression of TFAM [122], suggesting that defective damaged mtDNA removal is implicated in the pathogenesis of PD.

In conclusion, it is apparent that mitochondrial dysfunction and inflammasomes activation are critical emerging players in inducing and sustaining neuroinflammation during PD.

### 2.4. Neuroinflammation and Iron Homeostasis in PD

Interestingly, neuroinflammation is highly connected to iron accumulation in PD. Cytokines regulate the expression of iron homeostasis proteins, such as ferritin, DMT1 and hepcidin, while reactive oxygen and nitrogen species trigger a c-aconitase to the IRP1 switch with the consequent iron accumulation amplifying the neurotoxic effects of unresolved neuroinflammation.

Accordingly, NFκB promotes the expression of the iron importer DMT1 gene [123,124]. The nuclear translocation of NFκB occurs downstream of many cytokine receptors, such as the TNF receptor (TNFR) and the IL-1 receptor (IL-1R) (reviewed in [125]). Importantly, the nuclear translocation of NFκB is increased in the dopaminergic neurons of PD patients (9207126), which express high levels of TNFR [126]. Accordingly, TNF-α, IL-6 or the TLR4 agonist, LPS, directly increases both DMT1 mRNA and protein levels, generating iron accumulation in neurons and microglia [127,128].

Ferritin, which stores iron in a redox inactive form, is also regulated by inflammation. Ferritin is composed of 24 ferritin-H and ferritin-L subunits in cell-specific ratios. These subunits are not functionally interchangeable and are critical for maintaining iron homeostasis and protecting against iron overload. Ferritin-L has a higher capacity than ferritin-H to induce iron-core nucleation, whereas ferritin-H is superior in promoting iron oxidation [129]. Ferritin-H expression is regulated by the activation of the transcription factors NFκB [130,131] and NF-E2-related factor 2 (Nrf2) (reviewed in [132]).

In PD patients, the SNpc neurons have diminished ferritin levels, their being ferritin iron load higher than the ferritin from control patients [133]. Relatively low levels of ferritin and iron accumulation make DA neurons more susceptible to oxidative stress in the SNpc. Moreover, the ferritin-H/ferritin-L ratio in PD is also changed, with increased ferritin-H and decreased ferritin-L, reducing the capacity of ferritin to store iron [134].

Pro-inflammatory cytokines, such as IL-6 and IL-1β, induce hepcidin synthesis through the activation of the transcriptional factors STAT3 and NFκB (reviewed in [135]). Hepcidin is a small peptide hormone that acts as the master regulator of systemic iron homeostasis, which is expressed in several brain regions, including the cortex, hippocampus, amygdala, thalamus, hypothalamus, olfactory bulb, mesencephalon, cerebellum, pons and spinal cord [136,137,138], as well as in the endosomal structures in the reactive astrocytes and epithelial cells of the choroid plexus, colocalizing with FPN1 [139]. Hepcidin downregulates the iron exporter FPN1, with cell type-specific effects on brain iron homeostasis. Hepcidin expression triggers iron accumulation on neurons [128,140]. On the contrary, in astrocytes that participate in iron incorporation across the blood-brain barrier [141], hepcidin reduces blood-to-brain iron passage, thus reducing brain iron overload [142,143]. The overexpression of hepcidin in brain cells decreases neuronal loss and mitochondrial dysfunction in rotenone or 6-OHDA-injected mice [144]. Endogenous hepcidin levels in the cerebrospinal fluid of PD patients have not been evaluated until now, although an increase in serum levels has been observed [145], possibly triggered by inflammatory mediators or deregulated iron homeostasis.

Finally, inflammation mediators also regulate the IRE/IRP system. Proinflammatory cytokines IL-1β and TNF-α increase IRP1 levels, generating an increment of DMT1 and TfR1 protein levels, together with a decrease in FPN1, triggering iron accumulation in ventral mesencephalic neurons. These changes are abolished by the co-administration of the anti-oxidant N-acetylcysteine or the inducible nitric oxide synthetase inhibitor Nω-nitro-l-arginine methyl ester hydrochloride [146]. IRP1 displays a complex response to reactive oxygen/nitrogen species. Both NO and H_2_O_2_ disrupts the Fe-S cluster of the c-aconitase, turning it into IRP1 [147,148,149,150]. Accordingly, an increase in the iron labile pool is observed in NO or H_2_O_2_-treated cells [151].

In summary, neuroinflammation, mitochondrial dysfunction and iron accumulation are intertwined in a positive feedback loop that supports the development and progression of PD (Figure 1).

## 3. Current Therapeutic Approaches to PD

In addressing the pathophysiology of PD, an attractive hypothesis is the metal-based neurodegeneration hypothesis [152]. According to this hypothesis, redox-active metal ions, such as iron, together with mitochondrial dysfunction, generate ROS, which causes the peroxidation of membrane phospholipids, which in turn leads to the formation of reactive aldehydes. Reactive aldehydes and ROS modify α-synuclein, inducing its aggregation. Aggregated α-synuclein affects mitochondrial function, generating a positive loop of more ROS production and less ATP synthesis. From the therapeutic standpoint, it follows that multiple-task strategies targeting these events should provide more effective treatment to stop the progression of this disease.

Here, we will review finished clinical trials with published results that were designed to slow down the neurodegenerative process of PD, in particular, trials addressing the PD-associated pathological events of increased ROS production, iron overload, α-synuclein aggregation and inflammation. To this effect, we used the data base of the Clinicaltrials.gov (https://beta.clinicaltrials.gov: accessed on 13 December 2022) as a source. A comprehensive analysis of ongoing trials aimed either to decrease the symptoms or to delay/slow the progression of the disease is found in [153].

### 3.1. Clinical Trials Targeting Oxidative Stress

Several clinical trials have aimed to decrease the oxidative damage observed in PD by utilizing ubiquinol, the anti-oxidant form of Coenzyme Q10 (CoQ10). As mitochondrial Complex I dysfunction is a common trait in sporadic PD, treatment with CoQ10, intended to bypass Complex I dysfunction, could restore mitochondrial function [154].

A Meta-Analysis study of eight randomized controlled trials using CoQ10 (899 patients) concluded that CoQ10 was safe and well tolerated; however, no improvement of motor symptoms was observed compared to the placebo. The authors indicated that they cannot recommend CoQ10 for routine treatment of PD [155].

Overall, the clinical evidence indicates that supplementation with CoQ10 does not improve the motor symptoms of PD patients, perhaps with the exception of those with wearing-off resulting from years of using levodopa.

### 3.2. Clinical Trials Using Iron Chelation Therapy

A search in https://beta.clinicaltrials.gov: accessed on 14 December 2022 using Parkinson’s disease and iron chelation as keywords showed four finished clinical trials, only two of them with published results.

In clinical trial NCT01539837, reported by researchers from the Imperial College London, good tolerance to deferiprone was found in PD patients. Although the removal of excess iron in the dentate and caudate nucleus was found, the treatment with deferiprone had minimal effects on the symptoms of the disease [156].

A randomized pilot clinical trial tested 40 patients with early-stage PD treated with the iron chelator deferiprone (ClinicalTrials.gov Identifier NCT00943748). A dose of 30 mg/kg body weight per day, during a period of six months, resulted in decreased iron content in the substantia nigra, evaluated by T3 magnetic resonance. In addition, a smaller change in the UPDRS score in the deferiprone-treated group, compared to the placebo group, was also found. Nevertheless, once the treatment was suspended, iron accumulation reappeared, suggesting a reversal to the pathological state [157]. In a second report of the same study, the usefulness of the ferroxidase ceruloplasmin (CP) as a biomarker was emphasized, associating the low activity of this enzyme in Parkinson’s disease with iron overload in the substantia nigra [158]. It was found that after six to 12 months of deferiprone treatment, greater reductions in the substantia nigra iron levels and UPDRS motor scores were obtained in patients with higher serum and cerebrospinal fluid levels of CP-ferroxidase activity. A second stage of this project, under the acronym FAIRPARK II, enrolled 372 early-diagnosed PD patients who had never received levodopa. (https://clinicaltrials.gov/ct2/show/NCT02655315: accessed 11 January 2023). The patients were supplemented with of 15 mg of deferiprone per kilogram of body weight or with the placebo, twice daily, for 36 weeks. At week 36, the MDS-UPDRS score increased by 15.6 points (i.e., worsened) in the deferiprone group, compared with 6.3 points in the placebo group, despite a decrease in brain iron content in the deferiprone group [159]. Based on these findings, the authors concluded that deferiprone supplementation to early-diagnosed PD patients was associated with worse MDS-UPDRS scores, despite the decreased brain iron content.

In summary, the reported results on the iron chelation treatment of PD discussed above are not encouraging in terms of a possible slowdown of the disease progression.

### 3.3. Clinical Trials Targeting a-Synuclein Aggregation

A Phase II study (SPARK, NCT03318523) evaluated the effects of the monoclonal antibody BIIB054 (trade name Cinpanemab), which targeted aggregated forms of α-synuclein in participants with PD. BIB054 was administered every four weeks via intravenous (IV) infusion to adults with PD, at doses between 250 and 3500 mg. No effect on the MDS-UPDRS score at any dose tested was found. Due to “negative clinical trial findings”, the producing company, Biogen, discontinued the development of Cinpanemab as a medication intended to treat PD in 2021 (https://www.alzforum.org/therapeutics/cinpanemab: accessed 14 December 2022).

Overall, the finished clinical trials targeting α-synuclein aggregation by monoclonal antibody treatment have failed to produce evidence of an improvement of disease conditions. Nonetheless, a number of other studies targeting α-synucleinopathies, either in the recruiting step or in progress, may shed evidence that α-synuclein aggregation is indeed a therapeutic target in PD. These trials are found in https://beta.clinicaltrials.gov/search?patient=Parkinson%C2%B4s%20Disease%20%CE%B1-Synuclein&locStr=&distance=0&page=9: accessed 14 December 2022.

### 3.4. Clinical Trials Targeting Inflammation

There are no finished clinical trials that specifically target inflammation as a treatment for PD. Clinical trial NCT03462680 studied the effect of the daily supplementation of niacin (vitamin B3) on motor symptoms in PD patients. Based on the knowledge that niacin inhibits vascular inflammation and protects against endothelial dysfunction [160], niacin can be considered an anti-inflammatory drug. A six-month double-blind, placebo-controlled randomized study produced evidence that the UPDRS III scores significantly decreased in PD patients compared with the placebo group [161].

Following the recognized link between the intestinal environment and inflammation, a number of clinical trials are now in the recruiting step to evaluate the effects of microbiota intervention and of nutritional supplementation with anti- inflammatory compounds on a putative improvement of clinical parameters in PD patients. (https://beta.clinicaltrials.gov/search?patient=Parkinson%C2%B4s%20Disease%20inflammation&locStr=&distance=0&page=4: accessed 14 December 2022).

Overall, clinical interventions devoted to decreasing inflammatory conditions in PD could be a promising path to follow.

## 4. Multifunctional Drugs for the Treatment of PD

The lack of success observed in one target trial raises the need for drugs that attack multiple aspects of the PD neurodegenerative process. In addition to targeting the pathological aspects of the disease, the mitochondrial destination is also a desirable property of a putative drug as the mitochondrion can be viewed as a confined space in which both high concentrations of ROS and of iron coexist, which makes this organelle particularly prone to oxidative damage [103,104,162].

During the last two decades, several multifunctional agents have been reported to be effective in experimental models of PD. It must be considered that a treatment with a multitarget drug has advantages over the combination therapy of several single-target drugs. By reducing polypharmacy (i.e., the mixing of many drugs), multitarget drugs decrease the risk of adverse effects and the risk of drug-drug interactions [163]

Table 1 shows the basic characteristics of these agents, including their metal chelation characteristics, their capacity to act as anti-oxidants/free-radical scavengers, their route of administration and their blood-brain barrier permeability.

At present, there are several chemical nuclei that could be used for the design and development of new substances with a multi-target capacity. Below, we will analyze the active chemical groups and the functions they influence.

### 4.1. Derivatives of Phenols

**Figure 2:**
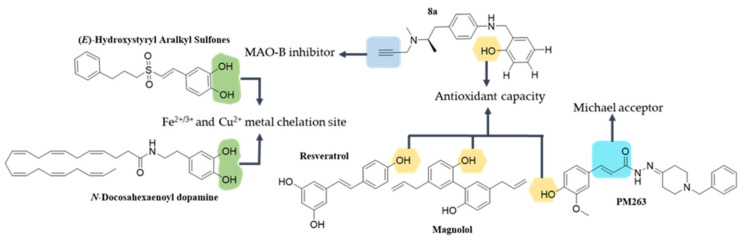
**Structure-function association of multifunctional phenolic derivatives.** Green shadowing shows iron chelation sites; yellow shadowing shows free radical scavenger sites.

Phenols are compounds with multiple activities that could play a key role in the development of new multi-target drugs for the treatment of neurodegenerative diseases. These include resveratrol, magnolol and PM263, among others. The association between the structure and function of putative neuroprotective phenolic derivatives is shown in Figure 2.

Resveratrol is a natural compound of the stilbene family that is present in appreciable concentrations in the skin of grapes and red wine. Cell studies show that resveratrol decreases the concentrations of ROS and NOS, and stimulates the increase in endogenous anti-oxidant systems, such as Nrf2, HO-1, SOD, CAT and GSH [164,191,192,193]. In neuroinflammation, resveratrol decreases the activity of the pro-inflammatory pathways and increases the levels of the anti-inflammatory cytokine IL-10 [194,195,196]. In autophagy, it induces the control of LKB1 and AMPK over mTOR, Beclin-1, LC3-II and p62 signaling [197,198], whereas in apoptosis, it exerts activity on Caspase-3, Caspase-9, Bax, Bcl-2 and SIRT-1/pAMPK [199,200]. These data indicate that resveratrol acts as a modulating agent of the various synergistic pathways involved in anti-oxidant, anti-inflammatory and anti-apoptotic activities.

Magnolol is a very particular hydroxylated biphenyl compound extracted from the Magnolia officinalis species. It exhibits inhibitory activity of the NF-κB and MAPK signaling pathways, which are associated with neuroinflammation and oxidative processes [165].

A group of multifunctional cinnamoyl-*N*-acylhydrazone-donepezil hybrid compounds were synthesized. From these, PM-263 was evaluated in in vitro models of neurodegenerative diseases [166]. Testing showed that PM-263 inhibits ROS generation, has moderate inhibitory activity towards acetylcholinesterase and effects the protection of cell viability against 6-OHDA-induced damage.

The structure of Compound 8a displays a potential site for MAO-B inhibition and functional groups with putative anti-oxidant activity and potential metal interaction sites [167]. Tested in cells, compound 8a inhibited MAO-B activity at submicromolar concentrations and presented outstanding anti-oxidant activity and chelation of Fe^2+^, Cu^2+^ and Zn^2+^ ions. In addition, it was observed that Compound 8a inhibited the Cu^2+^-induced aggregation of Aβ_1-42_ [167].

A group of (E)-hydroxystyryl aralkyl sulfone derivatives (compounds 5a to 5h) were synthesized and tested in their anti-oxidant and as neuroprotective activities. All of these compounds had a catechol group with potential anti-oxidant and metal chelating properties. The results showed free radical scavenging and neuroprotective effects through in vitro assays from neurotoxins such as H_2_O_2_, 6-OHDA and MPP^+^ [168]. In addition, compound 5h showed the highest anti-inflammatory capacity using the LPS-induced nitric oxide release model in BV2 microglia.

N-Docosohexaenoyl dopamine is a compound that presents a catechol group, favorable for exerting anti-oxidant activity and chelating ions such as Fe^2+^ and Cu^2+^ [201]. This compound is an endogenous bioactive lipid of the neurolipin family and shows neuroprotective activity at micromolar concentrations.

### 4.2. Derivatives of Polyphenols

**Figure 3:**
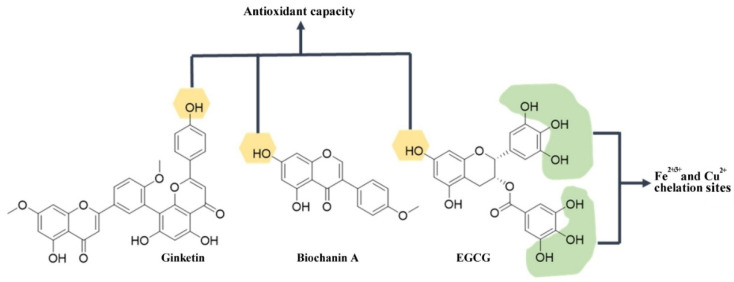
**Structure of flavonoids with neuroprotective activity.** Green shadowing shows iron chelation sites; yellow shadowing shows free radical scavenger sites.

Flavonoids are natural compounds with a wide range of health-promoting activities on diseases that include cancer, heart and neurodegeneration [202,203,204]. Widely characterized flavonoids with therapeutic potential include Ginkgetin, Biochanin A and EGCG. The association between the structure and function of these neuroprotective polyphenol derivatives is shown in Figure 3.

The bioflavonoid Ginkgetin, a compound isolated from the species *Ginkgo biloba* L., presented significant protection against MPP-induced damage, as represented by the decrease in reactive oxygen species, which correlates with the molecule’s multiple hydroxyl groups [170] (Figure 3). Ginkgetin inhibited cell apoptosis through the caspase-3/Bcl2/Bax pathway and had a stabilizing effect on SNpc tyrosine hydroxylase expression and on SOD activity in the striatum. In addition, this compound could chelate ferrous and ferric ions, indicating that the mechanism by which it exerts neuroprotective activity is through decreasing redox-active iron [170].

A compound that exhibits promising neuroprotective activity is the isoflavone Biochanin A (Figure 3), which is isolated from a variety of plants of the family Leguminosae (reviewed in [171]). Biochanin A has three hydroxyl groups (Figure 3) which are responsible for the anti-oxidant capacity [205,206], and it is possible that they also coordinate ferrous ions. Within the mechanism of neuroprotective activity, this compound blocks microglial activation and increases SOD, NADPH oxidase and glutathione peroxidase. In addition, it has a direct effect on decreasing NO, TNF-α, IL-1β and ROS concentrations in vitro, as well as other attributes associated with neuroprotection [205].

Another widely known compound is (−)-epigallocatechin-3-gallate (EGCG) [172], distributed in various plant species with high anti-oxidant power. This compound is characterized by its polyphenolic, ferrous and ferric ion chelating capacity (Figure 3). In this study [172], EGCG was the most abundant polyphenol of the polyphenol mixture tested. The administration of the mixture was found to reduce the α-synuclein concentration. Furthermore, it was concluded that this polyphenol mixture, where EGCG is relevant, had the ability to stabilize motor disturbances and α-synuclein aggregation.

### 4.3. Derivatives of Coumarins

**Figure 4:**
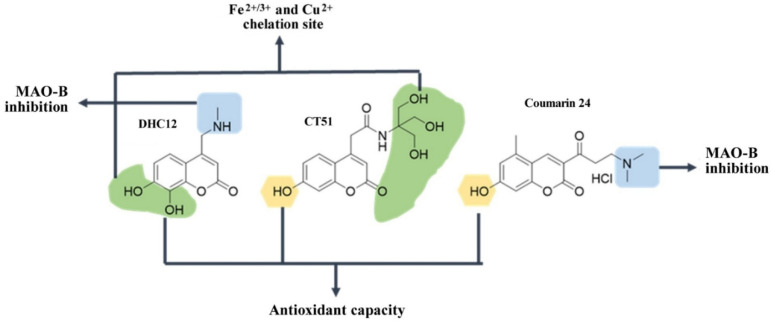
**Structure of coumarins with neuroprotective activity.** Green shadowing shows iron chelation sites; yellow shadowing shows free radical scavenger sites; blue shadowing shows MAO-B inhibition sites.

Our research group has developed the multi-white coumarin compounds, DCH12 [173] and CT51 [174] (Figure 4). In affinity studies of metals of biological interest, both compounds showed a chelation of ferric and ferrous ions. However, DHC12 showed a higher affinity constant compared to the values obtained from CT51. In addition, DHC12 also showed affinity for cuprous ion, a metal that can also mediate the Fenton reaction for the generation of the hydroxyl radical. In vitro studies with DHC12 concluded that it is a compound with chelating capacity that localizes to cytoplasm and mitochondria. In addition, DHC12 showed protection against lipid peroxidation, stabilized mitochondrial membrane potential and inhibited MAO-B activity (Figure 4).

In the in vivo studies, DHC12 showed a neuroprotective capacity in neurons of the SNpc. CT51 showed a potent capacity to inhibit radical species by chemical experiments based on EPR studies. In cells, CT51 was also distributed to the mitochondria, protected against lipid peroxidation and reduced the sustained release of calcium in the ryanodine receptor (RyR) upon an oxidation stimulus. However, unpublished results from our group indicated that CT51 is toxic to mice at putative therapeutic doses.

### 4.4. Derivatives of Quinolines and Quinolones

**Figure 5:**
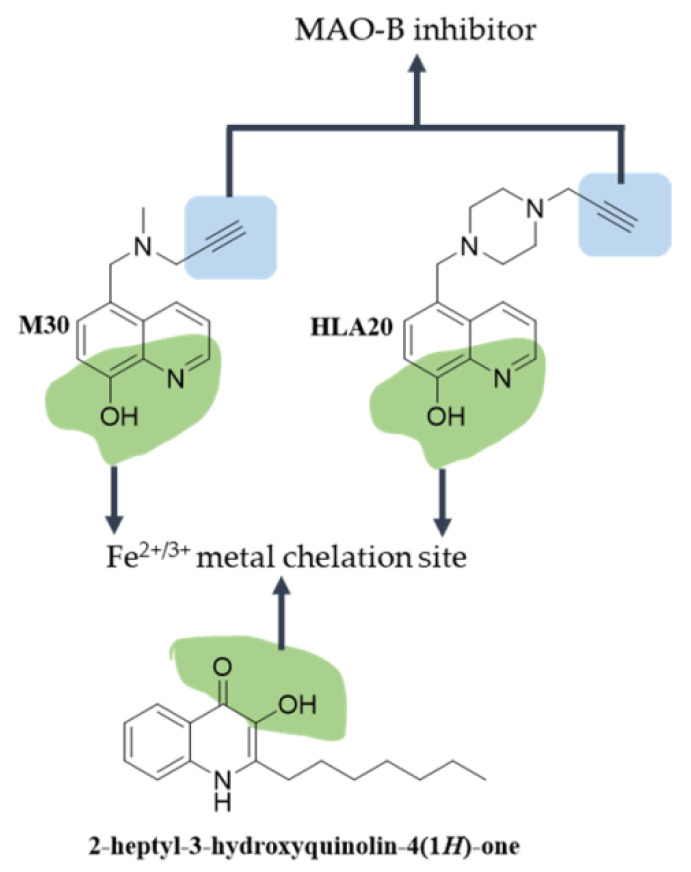
**Structure of quinolines and quinolones with neuroprotective activity.** Green shadowing shows iron chelation sites; blue shadowing shows MAO-B inhibition sites.

The quinoline derivatives M30 [176] and HLA-20 are versatile compounds characterized by a *N*- propargylamine MAO-B inhibition group and a metal (Fe^2+^, Fe^3+^, Cu^2+^, Zn^2+^) chelation site [177] (Figure 5).

M30 has neuroprotective activity in vitro, and its in vivo treatment results in an increase in the hypoxia inducible factor HIF-1 [207]. It differentially induces endothelial growth factor, erythropoietin, enolase-1, transferrin receptor, heme oxygenase-1, inducible nitric oxide nitric oxide synthase, glucose transporter 1, brain-derived neurotrophic factor, glial cell-derived neurotrophic factor, anti-oxidant enzymes (catalase, superoxide dismutase 1 and glutathione peroxidase). It has been shown to induce increased phosphorylation of protein kinases PKC and PKB/Akt and glycogen synthase 3 [207,208,209,210,211].

HLA-20 (propargyl hydroxyquinoline) exhibits a behavior similar to M30. It features a MAO-B inhibition and a metal chelation site. In vitro, it shows neuroprotective activity against glutamate-induced neurotoxicity and a reduction in ROS accumulation mediated by the inhibition of MAPK pathway activation (p38, ERK and JNK). Notably, it protects neuronal cells from apoptotic death [177,178,212,213,214].

Discarding the possible toxicity effects, it is apparent that M30 and its derivatives are serious potential drug candidates for PD treatment.

### 4.5. Derivatives of Piperazine

**Figure 6:**
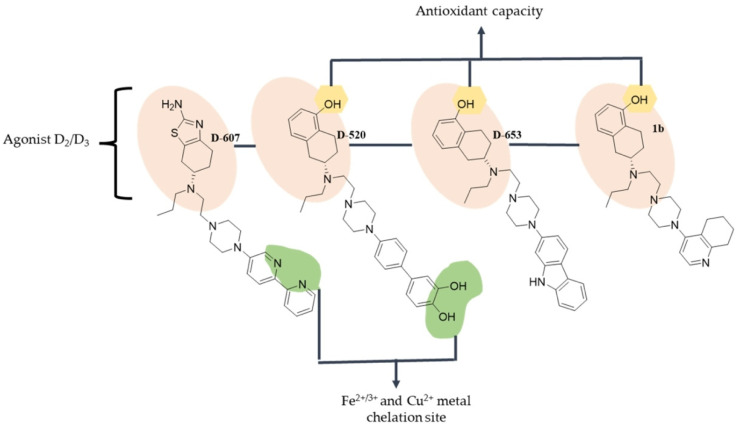
**Structure of piperazine with neuroprotective activity.** Green shadowing shows iron chelation sites; yellow shadowing shows free radical scavenger sites.

The piperazine derivatives D-607 [215], D-520 [180], D-653 [181] and 1b [182] are characterized as D2/D3 agonists. In addition, they have anti-oxidant and metal chelation features that allow them to be classified as multi-target drugs designed to treat PD (Figure 6).

D-607 has a ferrous and ferric ion chelating site consisting of a bipyridyl group [216], which also inhibits prolyl hydroxylase [217], and an anti-oxidant center consisting of 4,5,6,7-tetrahydrobenzo[d]thiazol-2-amine. Ligand receptor binding assays at dopamine D2/D3 receptors indicate very high affinity, showing in vivo activity in reserpinized PD. In vitro, it showed the protection of PC12 cells against 6-OHDA toxicity [215]. In addition, another study showed that, in vivo, it protects dopaminergic neurons from MPTP toxicity [215].

D-520 has a meta chelation site consisting of a catechol, which in turn can act as an anti-oxidant site in conjunction with a hydroxyl group. It also acts as a multifunctional dopamine D2/D3 receptor agonist. It inhibits the formation of Aβ aggregates in vitro and promotes the disaggregation of α-synuclein and Aβ aggregates in the Drosophila melanogaster model of Aβ1-42-dependent toxicity [180].

D-653 is a proposed multifunctional drug with a high agonist affinity on dopamine D2/D3 receptors, no identifiable chelating site, a carbazole ring and conditions to have anti-oxidant capacity as it has isolated hydroxyls. In the in vitro assays, D-653 showed reduction in oxidative stress induced by the neurotoxin 6-OHDA [181], whereas in the in vivo models, it showed potent activity to reverse hypo-locomotion compared to the clinically used compound, ropinirole [218]. These observations lead to the conclusion that D-653 exerts neuroprotective activity and is a candidate for the development of a substance for the treatment of PD.

Compound 19b [182], similarly to its predecessors, is a dopamine D2/D3 agonist. Although it does not have a clear chelation site, this compound showed chelating activity against iron and potent anti-oxidant activity [182]. In the in vivo models, 19b reversed the locomotor activity in reserpinized rats [182]. These observations lead to the conclusion that 19b is a candidate for drug development for the treatment of PD.

### 4.6. Derivatives of Pyrazine and Pyridones

**Figure 7:**
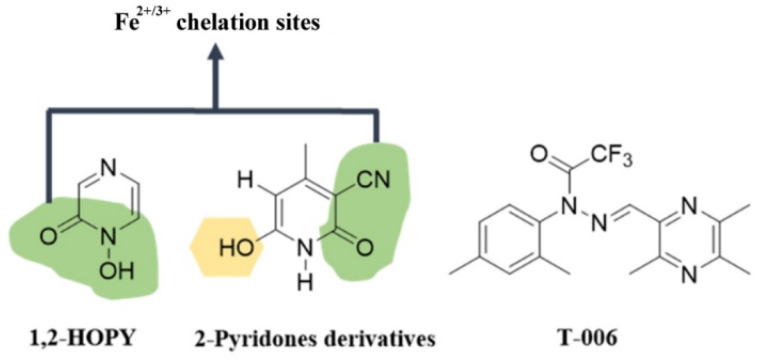
**Structure of pyrazine and pyridones with neuroprotective activity.** Green shadowing shows iron chelation sites; yellow shadowing shows free radical scavenger site. T-006 does not have a demonstrated metal chelation site nor anti-oxidant activity. Nonetheless, it has proven neuroprotective activity.

Deferiprone was one of the first Fe^2+^/Fe^3+^ chelators used in clinic to decrease the iron load in the blood [219]. This small bidentate molecule showed significant results on iron excretion when administered orally and peritoneally [220]. In recent years, alternative chelators have been proposed that have a significant effect in the treatment of PD based on the principle of simple heterocyclic chelators that permeate the BBB. Based on the above, work has been published on the development of molecules such as 1,2-HOPY [183,221]. This compound showed chelating activity against ferrous and ferric ions and also showed a free radical trapping capacity (Figure 7).

In a recent study, 24 compounds derived from 2-pyridone were synthesized [184]. Among these compounds, compound 6 (6-hydroxy-4-methyl-2-oxo-1,2-dihydropyridine-3-carbonitrile) showed the most promising results. This compound exhibited a metal chelation site and a hydroxyl group to which the reported anti-oxidant activity can be ascribed (Figure 7). It also demonstrated neuroinflammatory activity by reducing the production of NO, IL-6 and TNF-α. In vitro neuroprotective activity was also observed.

Compound T-006 [185,222] improved the mitochondrial membrane potential loss and energy metabolism, in vitro. In the in vivo experiments, the results showed that T-006 decreased the 6-OHDA-induced loss of tyrosine hydroxylase positive neurons, as well as dopaminergic nerve fibers in the striatum, and the functional impairments decreased after T-006 treatment in the 6-OHDA-injured animals. Its neuroprotective effects were attributed to the activation of the PKA/Akt/GSK-3β and CREB/PGC-1α/NRF-1/TFAM pathways (Figure 7).

### 4.7. Derivatives of Terpenoids

**Figure 8:**
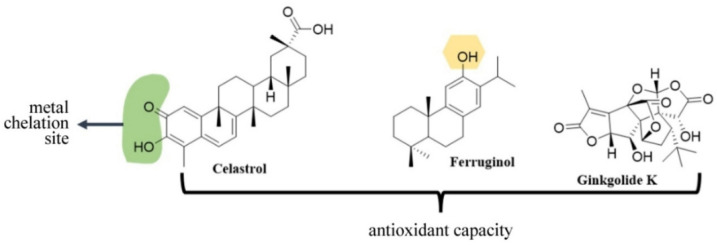
**Structure of Terpenoids with neuroprotective activity.** Green shadowing shows iron chelation sites; yellow shadowing shows free radical scavenger site. The APH-4 compound has anti-oxidant and metal chelating capacity; the latter not specified; In MT-20R the blue box shows the inhibition site for MAO-B.

Natural products have a wide variety of chemical nuclei, one of the most important being the terpenes. These substances can be classified according to the number of carbons into monoterpenes, sesquiterpenes, diterpenes, sesterpenes, triterpenes and tetraterpenes. They all exhibit diverse biological activities and have given rise to drugs for multiple biological targets. These includes Celastrol, Ferruginol and Ginkgolide K (Figure 8).

Celastrol [186] is a pentacyclic acid triterpene derived from oleanane. This compound has two active sites, the first of which is in ring 1, where it has a carbonyl and a hydroxyl functional group. These can exert chelating activity against various metals and display anti-oxidant activity, as well as the carboxyl group located in ring 5. This triterpene has anti-inflammatory and anti-oxidant capacities. Celastrol stabilized the motor deficits in an in vivo model of PD and showed inhibitory activity on NLRP3 inflammasome activation, as well as the dopaminergic neurodegeneration associated with the Nrf2-NLRP3-caspase-1 pathway [186].

Ferruginol and Ginkgolide K are diterpenes with an aromatic ring of tricyclic origin. Ferruginol is a diterpene with only one hydroxyl group that would confer anti-oxidant activity and has a possible coordination site with ferric ions. This compound demonstrated neuroprotective activities against apoptosis and MPTP-induced motor dysfunction in the in vitro and in vivo models of PD [187]. In addition, in SH-SY5Y cells transfected with the A53T α-synuclein mutant, Ferruginol attenuated the cytotoxicity induced by A53T α-synuclein. It promoted α-synuclein clearance neurons and inhibits its expression. It also inhibited the abnormal aggregation of the A53T α-synuclein mutant and the death of dopaminergic neurons [187]. The mechanism by which this process occurs has not yet been elucidated, but Ferruginol could eventually be a good candidate for PD treatment.

Ginkgolide K has a complex structure, with functional groups, such as hydroxyl and lactone rings, that can undergo metal-catalyzed opening processes, increasing its metal chelating capacity, an activity that has not yet been proven. Ginkgolide K exerted neuroprotective effects in MPTP-treated mice, improving signs of gait disease and protecting the dopaminergic neurons loss [188]. The compound exhibited immunomodulatory activity, including the induction of a microglial G2 phenotype and de-inflammation. Additionally, it promoted the co-localization GFAP/TH and GFAP/Nestin in astrocytes, an indication of a possible trans-differentiation of astrocytes into dopaminergic neurons in MPTP mice [188]. Its exact cellular and molecular mechanisms need to be further studied and ratified.

### 4.8. Others Molecules

**Figure 9:**
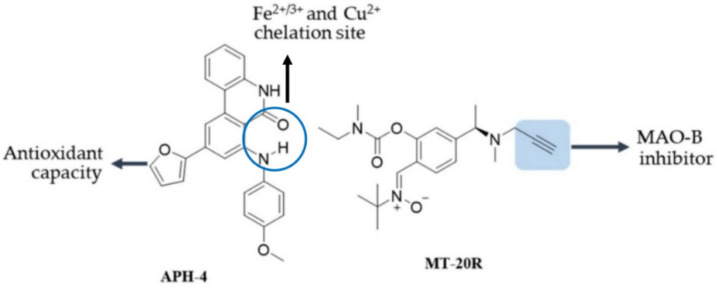
**Chemical cores in APH-4 and MT-20R with neuroprotective activity**. The APH-4 compound has anti-oxidant and metal chelating capacity; the latter not specified; In MT-20R the blue box shows the inhibition site for MAO-B.

APH-4 is a polycyclic molecule with amido, amino, methoxy and furan-based heterocycle functional groups (Figure 9).

According to a published report, this compound exhibits copper and iron ion chelating activity attributable to the neighboring amino and amide functional groups [189]. In addition, it induced anti-oxidant activity through the activation of the Nrf2 pathway and neuroprotective activity and reversed, in a concentration-dependent manner, the cytotoxic effects induced by Fe^2+^, Cu^2+^ or Zn^2+^ in the SN56 neuronal cells.

MT-20R [190] is a multifunctional compound of simple design that has not been studied for chelating activity, but has been designed to inhibit MAO-B both in vitro and in vivo. In the MPTP mice model of PD, MT-20R decreased the TH-positive DA neurons loss in the SNpc and improved the motor deficits [190]. In addition, it was observed that Bcl-2 expression was significantly enhanced, Bax and Caspase 3 expression was decreased and the AKT/Nrf2/HO-1 signaling pathway was activated. MT-20R also demonstrated hydroxyl radical and peroxynitrite radical scavenging capacities [190]. These properties make MT-20R yet another drug candidate for the treatment of PD.

It follows that a number of multi-functional compounds have the potential to be used as multi-target drugs for the treatment of PD and other neurodegenerative diseases that have iron accumulation, inflammation, and oxidative damage components. In continuing our analysis, we then proceeded to evaluate the putative drug-likeness of these compounds.

## 5. Drug-Likeness Prediction of the Multifunctional Compounds

The SwissADME program (http://www.swissadme.ch/: accessed 20 September 2022) was used to explore and achieve an in-silico prediction of the drug rating of the compounds used in this study, using different molecular descriptors [223] (Table 2). It must be considered that in-silico studies are just a reference regarding pharmacokinetic, drug-likeness, lipophilicity and other putative properties of a compound that do not replace the actual experimental testing.

It should be noted that most of the compounds comply with the main rules for compounds with the characteristics of a central nervous system drug. Only compounds 8a (4), D-607 (16), D-520 (17), D-653 (18) and Ferruginol (24) do not comply with one of the main Lipinski rules.

**Figure 10:**
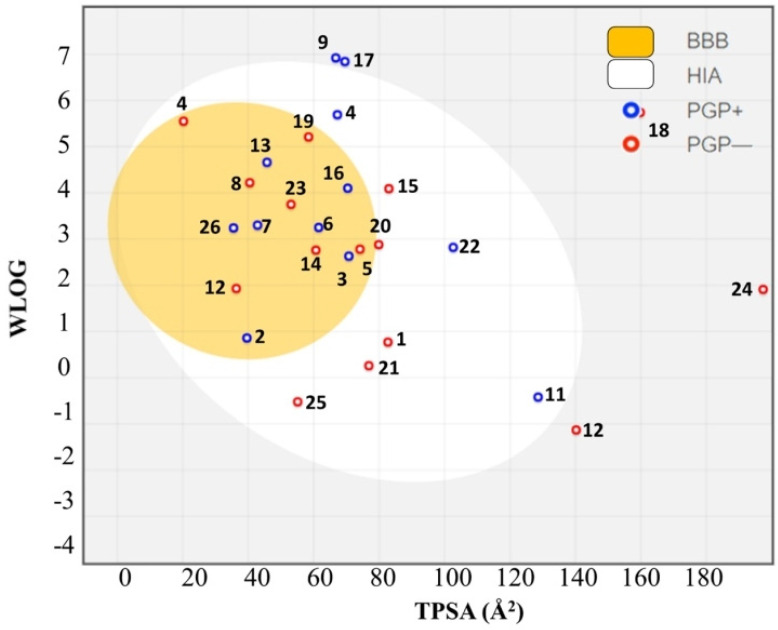
Predictive human intestinal absorption (HIA) model and blood-brain barrier permeation (BBB) method (boiled-egg plot) of the 27 compounds. Resveratrol (**1**), Magnolol (**2**), PM263 (**3**), 8a (**4**), (E)-Hydroxystyryl Aralkyl Sulfones (**5**), N-Docosahexaenoyl dopamine (**6**), Ginkgetin (**7**), Biochanin A (**8**), EGCG (**9**), DHC12 (**10**), CT51 (**11**), Coumarin 24 (**12**), M30 (**13**), HLA20 (**14**), 2-heptyl-3-hydroxyquinolin-4(1H)-one (**15**), D-607 (**16**), D-520 (**17**), D-653 (**18**), 1b (**19**), 1,2-HOPY (**20**), 2-Pyridones derivatives (**21**), T-006 (**22**), Celastrol (**23**), Ferruginol (**24**), Ginkgolide K (**25**), MT-20R (**26**) and APH-4 (**27**).

In the predictive study of the 27 compounds used in this review, the herbal egg model [224] was used to intuitively evaluate the passive gastrointestinal absorption (HIA) and BBB penetration, by means of lipophilicity (WLOG; Log Po/w) and TPSA calculations of the low molecular weight compounds (Figure 10). When evaluating the compounds for predictive BBB permeability, only 15 of the 27 compounds analyzed fulfilled the requisites.

Egg analysis showed that compounds with neuroprotective activity Magnolol (2), PM263 (3), *N*-Docosahexaenoyl dopamine (6), Ginkgetin (7), M30 (13), D-607 (16) and APH-4 (26), and found within the egg yolk, passively cross and are highly absorbable at the BBB, while compounds 8a (4), CT51 (11) and T-006 (22) egg-yolk zone are highly absorbable in the gastrointestinal tract. Compounds EGCG (9), D-520 (17), D-653 (18) and Ferruginol (24) are not absorbed and are not permeable to the BBB because they are outside the range of the graph. The compounds dotted in red—(*E*)-Hydroxystyryl Aralkyl Sulfones (5), Biochanin A (8), Coumarin 24 (12), HLA20 (14), 19b (19), 1,2-HOPY (20) and Celastrol (23)—penetrate the brain and are not subject to active efflux.

In summary, our structure-function analysis, together with the drug-likeness prediction, which importantly includes BBB permeability, identified compounds Resveratrol, Magnolol, PM263, Coumarin 24, M30, HLA20, D-607, compound 19b, 1,2-HOPY, Celastrol and APH14 as suitable multi-target drug candidates for the treatment of PD. However, the validity of any of these putative drugs must be corroborated in clinical trials.

## 6. Conclusions

PD is the second most common age-related neurodegenerative disorder, after Alzheimer’s disease, with ever-expanding health, social and economic impacts as result of increased longevity. In the etiology of PD, multiple processes converge, where mitochondrial dysfunction is a key factor in SNpc neuron death. The mitochondria is the center of the oxidative metabolism and the main site of continuous ROS production. Mitochondrial dysfunction triggers several interlinked processes, such as iron accumulation, oxidative stress, α-synuclein aggregation and neuroinflammation, that accelerate the neurodegenerative process. This complex interplay of mechanisms involved in the neurodegenerative process anticipates the reduced long-term success of therapeutic interventions that address single targets. Accordingly, several clinicals trials that have focused on one-target strategies have failed to stop the progression of the disease. Therefore, a multi-target approach that deals with several aspects of the neurodegenerative process simultaneously may be a better approach to stop the progress of the disease. Here, we propose several multifunctional agents with the potential to become a disease-modifying drugs for PD, affecting the underlying pathophysiology of the disease and possessing beneficial clinical outcomes.

## Figures and Tables

**Table 1:** Biological properties of multifunctional agents that could be used for the treatment of PD.

Compound	Active Chemical Groups	Metal Chelation	ROS Neutralization	Dopamine Metabolism ^#^	Mitochondria Tropism	Anti-Inflammation	In Vivo Effectiveness	Reference
Resveratrol	Phenol	Not tested	Yes	Not tested	Not tested	Yes	Yes	[164]
Magnolol	Phenol	Not tested	Yes	No	No	Yes	Yes	[165]
Cinnamoyl-*N*-Acylhydrazone-Donepezil Hybrids (PQM263)	Catechol and	No tested	Yes	Not tested	Not tested	Not tested	Not tested	[166]
Clioquinol-selegiline hybrids (compound 8a)	amino-hydroxyl	Cu^2+^; Fe^2+^; Zn^2+^	Yes	Yes	Not tested	Not tested	Yes	[167]
(E)-Hydroxystyryl Aralkyl Sulfones	Catechol	Not tested	Yes	Yes	Not tested	Yes	Not tested	[168]
*N*-Docosahexaenoyl Dopamine	Cathecol	Not tested	Yes	Yes	Not tested	Yes	Not tested	[169]
Ginkgetin	Enol and phenol	Fe^2+^	Yes	Not tested	Not tested	Yes	Yes	[170]
Biochanin A	Phenol	Not tested	Yes	Yes	Not tested	Yes	Yes	[171]
Epigallocatechin gallate (EGCG)	Catechol	Cu^2+^; Fe^3+^; Al^3+^; Mn^2+^	Yes	No	Not tested	Not tested	Yes	[172]
7,8-Dihydroxycoumarin derivative (DHC12)	Catechol	Cu^2+^∼ Fe^2+^ > Zn^2+^ > Fe^3+^	Yes	Yes	Yes	Not tested	Yes	[173]
Coumarin–tris hybrid (CT51)	2-amino-2-(hydroxymethyl)propane-1,3-diol (TRIS) and carbonyls	Fe^2+^ > Fe^3+^	Yes	Not tested	Yes	Not tested	Not tested	[174]
Coumarin Mannich base derivatives	No Present	Not tested	No	Yes	Not tested	Yes	Yes	[175]
Hydroxyquinoline-propargyl hybrids (M30)	quinolin-8-ol	Fe^3+^ > Cu^2+^ > Zn^2+(19b)^	No	Yes	Not tested	Not tested	Yes	[176]
Hydroxyquinoline-propargyl piperazine hybrids (HLA20)	quinolin-8-ol	Fe^3+^ > Cu^2+^	Yes	Yes	Not tested	Not tested	Yes	[177]
2-heptyl-3-hydroxy-4-quinolone	3-hydroxy-4-quinolone	Not tested	Yes	Not tested	Not tested	Not tested	Not tested	[178]
Dipyridyl-D2R/D3R agonist hybrids (D-607)	2,2′-Bipyridyl	Fe^2+^ >>> Fe^3+^	No	Yes	Not tested	Not tested	Yes	[179]
Cathecol-D2/D3 agonist (D-520)	Catechol	No tested	No	Yes	Not tested	Not tested	Yes	[180]
Carbazole- D2/D3 agonist hybrids (D-653)	No present	No tested	Yes	Yes	Not tested	Not tested	Yes	[181]
Piperazine–8-OH-quinolone hybrids (19b)	quinolin-8-ol	Fe^2+^; Fe^3+^	No	No	Not tested	Not tested	Yes	[182]
1-hydroxypyrazin-2(1H)-one (1,2-HOPY)	*N*-hydroxy-*N*-methylacetamide	Fe^3+^	Not tested	Yes	Not tested	Not tested	Not tested	[183]
2-pyridones derivatives	2-pyridones	Not tested	Yes	Not tested	Not tested	Yes	Not tested	[184]
Tetramethylpyrazine (T-006)	hydrazineylidene-pyrazine	Not tested	Yes	Yes	Not tested	Not tested	Yes	[185]
Celastrol	2-hydroxycyclohexanone	Not tested	Yes	Yes	Not tested	Yes	Yes	[186]
Ferruginol	Phenol	Not tested	Not tested	Yes	Not tested	Not tested	Yes	[187]
Ginkgolide K	Ascorbic Acid Derivatives	Not tested	Yes	Yes	Not tested	Yes	Yes	[188]
7-Aminophenanthridin- 6(5H)-one (APH-4)	8-aminoisoquinolin-1(2*H*)-one	Fe^2+^, Cu^2+^	Yes	Not tested	Not tested	Not tested	Not tested	[189]
Ladostigil derivative (MT-20R)	No	Not tested	No	Yes	Not tested	Not tested	Yes	[190]

^#^ Include D2/D3 agonism and modification of MAO-B activity.

**Table 2:** In silico prediction of drug-likeness *.

	Compound	MW	RB	HBA	HBD	MR	TPSA	Log P	Lipinski Violations	BBB Permeability
1	Resveratrol	221.21	2	5	3	59.10	82.70	0.80	0	Yes
2	Magnolol	281.35	3	4	1	92.05	39.60	1.90	0	Yes
3	PM263	310.75	4	5	1	82.84	70.75	1.23	0	Yes
4	Compound 8a	286.45	1	1	1	91.63	20.23	5.26	1	No
5	Compound 5h	367.44	10	5	2	107.96	74.16	2.84	0	Yes
6	*N*-Docosahexa-enoyl dopamine	373.49	9	4	0	112.18	61.53	2.95	0	Yes
7	Ginkgetin	448.64	7	4	1	143.84	42.84	4.08	0	Yes
8	Biochanin A	266.33	5	2	2	84.14	40.46	4.25	0	Yes
9	EGCG	428.6	2	4	3	126.90	66.76	5.29	0	No
10	DHC12	382.41	4	3	2	115.81	67.26	4.43	0	No
11	CT51	406.38	1	9	2	91.62	128.59	0.58	0	No
12	Coumarin 24	323.3	7	7	5	80.03	140.23	-0.31	0	Yes
13	M30	482.66	7	3	2	157.97	45.74	4.89	0	Yes
14	HLA20	228.24	2	3	3	67.88	60.69	2.48	0	Yes
15	2-heptyl-3-hydroxyquinolin-4(1*H*)-one	318.39	6	4	2	88.45	82.98	3.00	0	No
16	D-607	501.66	8	5	3	158.09	70.41	4.29	1	Yes
17	D-520	449.62	17	3	3	141.37	69.56	4.29	1	No
18	D-653	566.51	5	10	4	155.91	159.80	4.34	1	No
19	19b	364.36	5	7	0	94.44	58.45	3.94	0	Yes
20	1,2-HOPY	284.26	2	5	2	78.46	79.90	2.44	0	Yes
21	2-Pyridones derivatives	150.13	0	3	2	38.77	76.88	0.45	0	No
22	T-006	477.67	8	5	1	147.69	102.65	3.16	0	No
23	Celastrol	259.34	6	2	2	80.40	53.09	3.70	0	Yes
24	Ferruginol	458.37	4	11	8	112.06	197.37	1.01	1	No
25	Ginkgolide K	112.09	0	3	1	26.04	55.12	-0.35	0	No
26	APH-4	308.42	7	2	2	97.12	35.50	3.58	0	Yes
27	MT-20R	226.27	3	3	1	69.02	36.36	2.07	0	No

*: MW: molecular weight; RB, number of rotatable bonds; HBA, number of hydrogen bond acceptors; HBD, number of hydrogen bond donors; MR: molar refractivity; TPSA: topological polar surface area; Log Po/w: consensus octanol/water partition coefficient; BBB, blood-brain barrier.
